# Effects of Mo addition on crack tip opening displacement (CTOD) in heat affected zones (HAZs) of high-strength low-alloy (HSLA) steels

**DOI:** 10.1038/s41598-018-36782-6

**Published:** 2019-01-18

**Authors:** Seok Gyu Lee, Bohee Kim, Woo Gyeom Kim, Kyung-Keun Um, Sunghak Lee

**Affiliations:** 1 Center for Advanced Aerospace Materials Pohang University of Science and Technology, Pohang, 790–784 Republic of Korea; 2 Steel Products Research Group 1 Technical Research Laboratories, POSCO, Pohang, 790–785 Republic of Korea

## Abstract

Effects of Mo addition on microstructures and crack tip opening displacement (CTOD) in heat affected zones (HAZs) of three high-strength low-alloy (HSLA) steels were investigated in this study, and the correlation between them was explained by fracture mechanisms related with martensite-austenite constituent (MA) characteristics. The coarse-grained HAZ (CGHAZ) consisted of acicular ferrite (AF), granular bainite (GB), and bainitic ferrite (BF), whereas the inter-critically heated HAZ (ICHAZ) consisted of quasi-polygonal ferrite (QPF), GB, and MA. Since Mo promoted the formation of GB, BF, and MA and prevented the formation of AF and QPF, the CTOD decreased in both HAZs with increasing Mo content. According to the interrupted three-point bending test results of the ICHAZ where many MAs were distributed in the QPF or GB matrix, many voids were observed mainly at MA/QPF interfaces, which implied that the void initiation at the interfaces was a major fracture mechanism. The atomic probe data of MAs indicated the segregation of C, Mn, Mo, and P at MA/QPF interfaces, which could result in the easy MA/matrix interfacial debonding to initiate voids. Thus, characteristics of MA/QPF interfaces might affect more importantly the CTOD than the MA volume fraction or size.

## Introduction

Fracture toughness of heat affected zones (HAZs) is generally deteriorated in conventional high-strength low alloy (HSLA) steels by the formation of undesirable microstructural parameters induced from complicated heat cycles during welding processes^1–4^. The HAZs formed by single-pass heat cycles are classified into coarse-grained HAZ (CGHAZ), super-critically heated HAZ, inter-critically heated HAZ (ICHAZ), and sub-critically heated HAZ by peak temperatures of welding heat cycles. Among these HAZs, it is known that the CGHAZ and ICHAZ have the low fracture toughness because of large prior austenite grains and hard crack-susceptible martensite-austenite (MA) constituents, respectively^5–7^.

The fracture toughness of the HAZs is affected by alloying elements^8–11^. Mo is often added for enhancing strengths in the HSLA steels made by thermo-mechanically-controlled process (TMCP), whose microstructures are various low-temperature-transformed bainitic ones, *e.g*., bainitic ferrite (BF), granular bainite (GB), acicular ferrite (AF), and quasi-polygonal ferrite (QPF). The HAZ microstructures formed after the welding become more complicated, and their volume fractions significantly affect the fracture toughness.

In Mo-containing steels, Mo generally promotes activate the formation of MAs^10^, and thus its optimization is needed by understanding detailed fracture mechanisms. Particularly in the ICHAZ mixed with QPF and GB microstructures, hard MAs deteriorate the fracture toughness, and their crack susceptibility is varied with their hardness and MA/matrix interfacial features. However, fracture mechanisms related with cracking of MAs or void initiation at MA/matrix interfaces remain to be clarified by correlating the fracture toughness and fracture mechanisms.

Crack tip opening displacement (CTOD) used for the fracture toughness evaluation of offshore-application HSLA steel HAZs was measured in this study, and then were correlated with volume fractions of microstructures of BF, GB, AF, QPF, and MA in thermally-simulated HAZ specimens of three HSLA steels (S450~S500 grade, yield strength; 450~500 MPa) whose Mo content was varied. The steels containing 0.002%, 0.194%, and 0.350% Mo are referred to as ‘LM’, ‘MM’, and ‘HM’, respectively, for convenience. Optical and scanning electron microscope (SEM) observations along with electron back-scatter diffraction (EBSD) analyses were used for identifying and quantizing the complicatedly-mixed microstructures in the CGHAZ and ICHAZ. Interrupted three-point bending tests, nano-indentation, and 3-dim. atom probe (3-d AP) analyses were also conducted for examining characteristics of crack-susceptible MAs and their effects on crack initiation and propagation. Then, effects of Mo addition on microstructural evolution and CTOD of the HAZs were discussed to improve them further.

## Results

### Microstructures of coarse-grained HAZ (CGHAZ)

**Figure 1:**
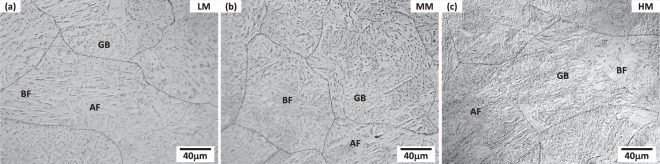
Optical micrographs of the simulated CGHAZ of the (**a**) LM, (**b**) MM, and (**c**) HM steels. The CGHAZs basically consist of AF, BF, and GB, and contain coarse packets and prior austenite grain boundaries whose sizes are larger than 40 μm. AF is a needle-shaped fine ferrite, and is grouped by packets having high-density interior dislocations. GB consists of relatively coarse packets, inside which island-shaped MA constituents are present. BF contains coarse packets consisted of parallel low-angle boundary substructures with fine secondary phases at the boundaries.

Optical micrographs of the CGHAZ of the LM, MM, and HM steels are shown in Fig. 1(a–c). They show coarse packets and prior austenite grain boundaries whose sizes are larger than 40 μm, and basically consist of AF, GB, and BF whose microstructures can be classified by their morphologies and features^8,12–23^, as marked in Fig. 1(a–c). AF is a needle-shaped fine ferrite, and is grouped by packets having high-density interior dislocations and high-angle boundaries which are effectively resistant to the cleavage crack propagation on {100} planes, thereby showing a good strength-toughness combination^26–28^. GB consists of relatively coarse packets, inside which island-shaped MA constituents are present to work as crack initiation sites and to deteriorate fracture toughness^8,22–25^. BF contains coarse packets consisted of parallel low-angle boundary substructures with fine secondary phases at the boundaries^8,12,22–25^.

**Figure 2:**
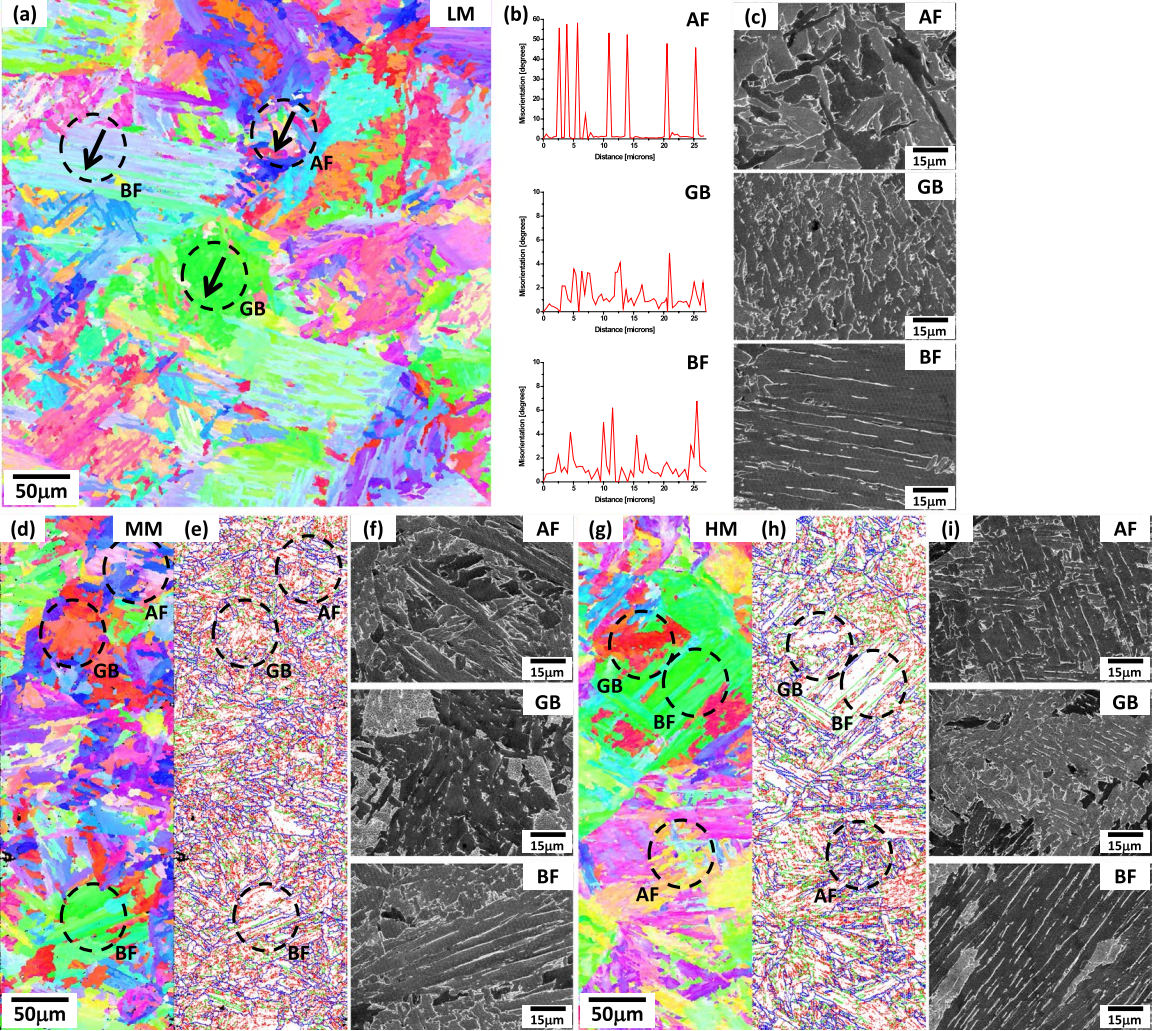
(**a**,**d**,**g**) EBSD inverse pole figure (IPF) map, (**b**,**e**,**h**) misorientation profiles and (**c,f,i**) SEM micrographs of AF, GB, and BF areas (black arrow marks in (**a**,**d**,**g**)) for the CGHAZ of the LM, MM, and HM steel. Most of boundaries of AF are high-angled ones (misorientations; 50~60 deg), and their spacings are small. Packets of GB are coarse (size; about 25 μm), and packet boundaries are irregular. Coarse packets of BF are defined by boundaries of 15-deg misorientations. These micrographs correspond with the microstructural classification of AF, GB, and BF, as aforementioned in the above paragraph.

An EBSD inverse pole figure (IPF) map and misorientation profiles of AF, GB, and BF areas (black arrow marks in Fig. 2(a)) for the CGHAZ of the LM steel are shown in Fig. 2(a,b). Most of boundaries of AF are high-angled ones (misorientations; 50~60 deg), and their spacings are small. Packets of GB are coarse (size; about 25 μm), and packet boundaries are irregular, inside which substructures are well developed. Coarse packets of BF are defined by boundaries of 15-deg misorientations (Fig. 2(b)). SEM micrographs of AF, GB, and BF areas of Fig. 2(a) (black-dashed-circle marks) are shown in Fig. 2(c). These micrographs correspond with the microstructural classification of AF, GB, and BF, as aforementioned in the above paragraph. The MA is hardly found in the LePera-etched CGHAZ^29^. These microstructural definitions combined with EBSD data are useful for classifications of AF, GB, and BF.

EBSD IPF and grain boundary maps and SEM micrographs of the CGHAZ of the MM and HM steels are shown in Fig. 2(d–i). AF, GB, and BF are defined again by the above microstructural classification method, and their volume fractions, along with effective grain sizes (*i.e*., prior austenite grain size) are measured, as shown in Supplementary Table 2. The AF fraction decreases in the order of the LM, MM, and HM steels (as the Mo addition increases), whereas the GB fraction shows an opposite trend of AF. The Mo addition reduces the diffusivity of carbon, delays the recrystallization of ferrite, and depresses the bainitic transformation^30,31^. According to this Mo addition effect, the bainitic transformation starts at the much lower temperatures to promote the formation of GB and BF at the lower transformation temperature than that of AF. With respect to the volume fraction data of microstructures in consideration of the CTOD data, thus, the Mo addition provides a detrimental effect on critical CTOD in the CGHAZ.

### Microstructures of inter-critically heated HAZ (ICHAZ)

**Figure 3:**
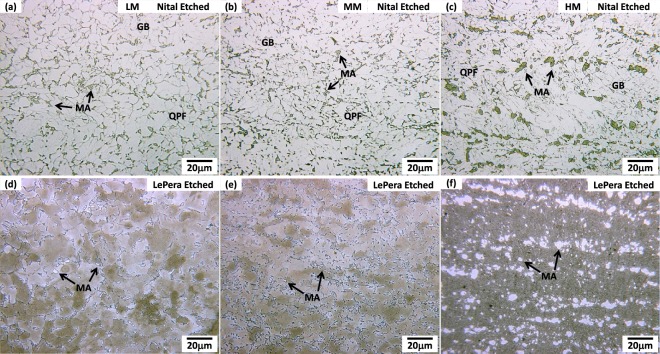
Optical micrographs of the simulated ICHAZ etched in (**a**–**c**) 1% nital solution and (**d**,**f**) LePera solution^29^ for the LM, MM, and HM steels. The ICHAZ is mainly composed of QPF and GB microstructures. Since MAs are white-colored, whereas other microstructures (QPF and GB) are dark-brown-colored, MA can be differentiated from QPF or GB, and its volume fraction can be measured. The average MA volume fraction is 4.2% in the LM steel, and tends to increase in the order of the MM and HM steels.

Optical microstructures of the ICHAZ of the three steels are shown in Fig. 3(a–c). They are mainly composed of QPF and GB microstructures. Since QPF is formed at a faster cooling rate and a lower temperature than the polygonal ferrite, its grain boundary shape becomes irregular^12^. Optical micrographs of LePera-solution-etched ICHAZs are shown in Fig. 3(d–f). Since MAs are white-colored, whereas other microstructures (QPF and GB) are dark-brown-colored, MA can be differentiated, and its volume fraction can be measured. The average MA volume fraction is 4.2% in the LM steel, and tends to increase in the order of the MM and HM steels. Most of MAs of the HM steel lie in a band shape along the rolling direction.

**Figure 4:**
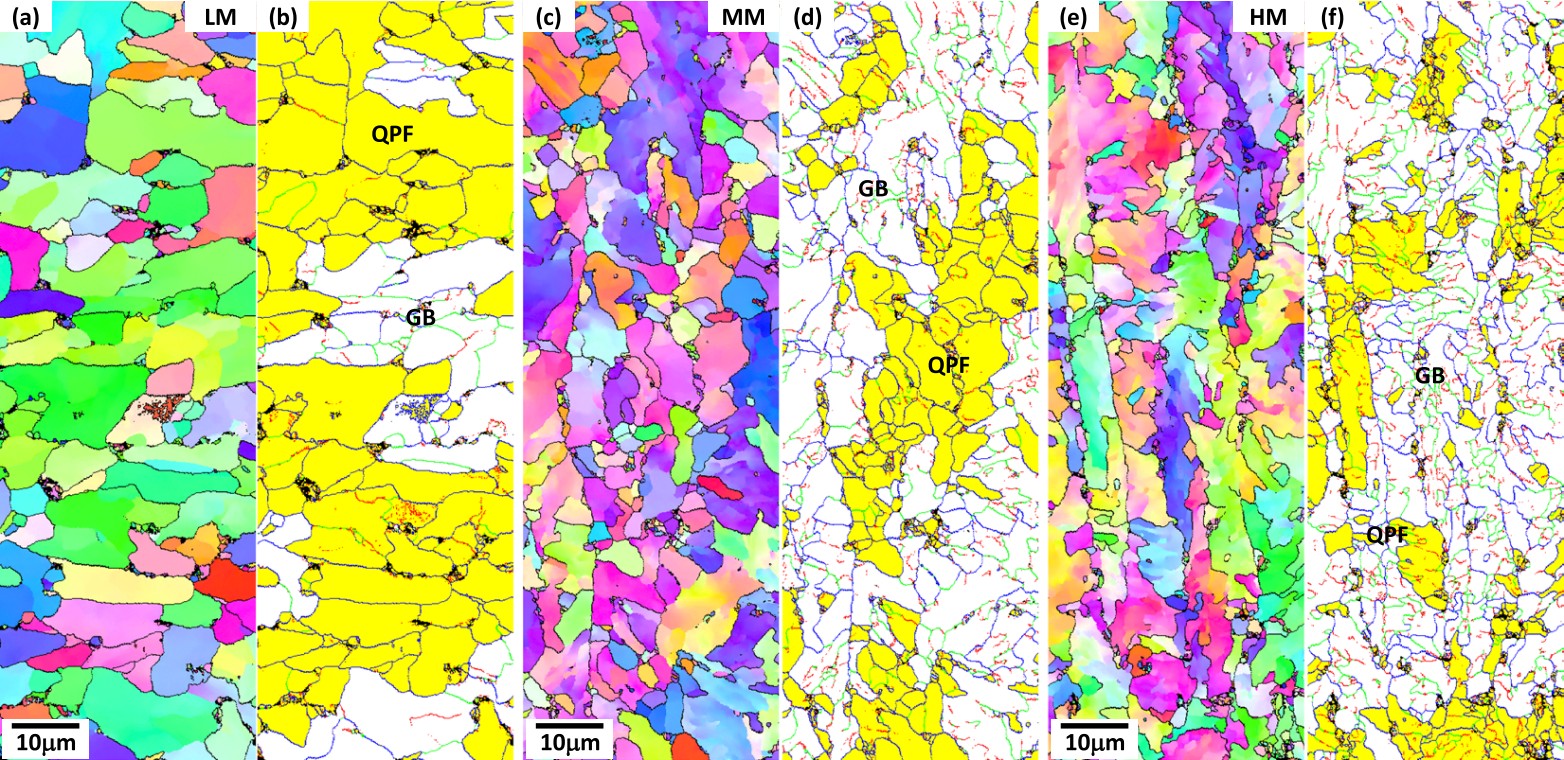
EBSD (**a**,**c**,**e**) inverse pole figure (IPF) and (**b**,**d**,**f**) grain orientation spread (GOS) maps of the ICHAZ of the LM, MM, and HM steels. The GOS maps show an extent of average misorientation between various datum points inside a grain. An angle of 15 deg is selected as a misorientation for defining grain boundaries, and grains having misorientations of 5 deg or smaller are defined as QPFs (yellow-colored areas in (**b**,**d**,**f**)).

Figure 4(a,c,e) shows EBSD IPF maps of the ICHAZ. It is confirmed again from microstructural morphologies that the ICHAZ is composed of QPF and GB microstructures. In order to differentiate QPF and GB, grain orientation spread (GOS) maps, which show an extent of misorientations between various datum points, are used for analyses of substructures, as shown in Fig. 4(b,d,f). Grain boundaries are defined by a misorientation angle of 15 deg, and grains whose misorientations are smaller than 5 deg are defined as QPFs (yellow-colored areas in Fig. 4(b,d,f))^32^. According to this GOS category, QPFs are differentiated from GBs because QPFs hardly develop substructures. The volume fraction of QPF based on the GOS analysis data is 73.3% in the LM steel, and decreases in the order of the MM and HM steels. The fractions of QPF, GB, and MA are shown in Table S2. The fractions of GB and MA increase as the Mo content increases, whereas that of QPF decreases.

### Crack tip opening displacement (CTOD) test results of simulated HAZs

Table S3 shows the room-temperature tensile test data of the HSLA steels. The yield and tensile strengths increase with increasing Mo content, whereas the elongation decreases, which shows the Mo addition effect on strengthening.

The CTOD test results of the CGHAZ and ICHAZ of the three steels are shown in Supplementary Table 4. At −40~−80 °C, the average critical CTOD is 0.43 mm in the CGHAZ of the LM steel, and decreases in the order of the MM and HM steel, which shows the opposite trend of the room-temperature strengths. At the other temperatures, trends of CTOD are similar to those of −40 °C, while the CTOD decreases as the test temperature decreases. In the ICHAZ, the CTOD tends to decrease as the Mo content increases, like in the CGHAZ.

### Crack initiation and propagation behavior in ICHAZ

Interrupted three-point bending tests were performed at −40~−80 °C by using sharp-notch-introduced rectangular-bar specimens (same to CTOD specimen dimensions). Since the plastic zone size is very small at the crack tip of the CTOD specimen, an electrical discharge machine (EDM) notch (notch depth; 3 mm, tip radius; ~200 μm) was inserted to the specimen. Typical load-displacement bending curves of the LM and MM steels are shown in Supplementary Fig. 1. Based on these curves, the bending test was stopped before the specimen fracture (red arrow marks), and then the specimen was cross-sectioned to observe the sharp-notch-tip area by an SEM.

**Figure 5:**
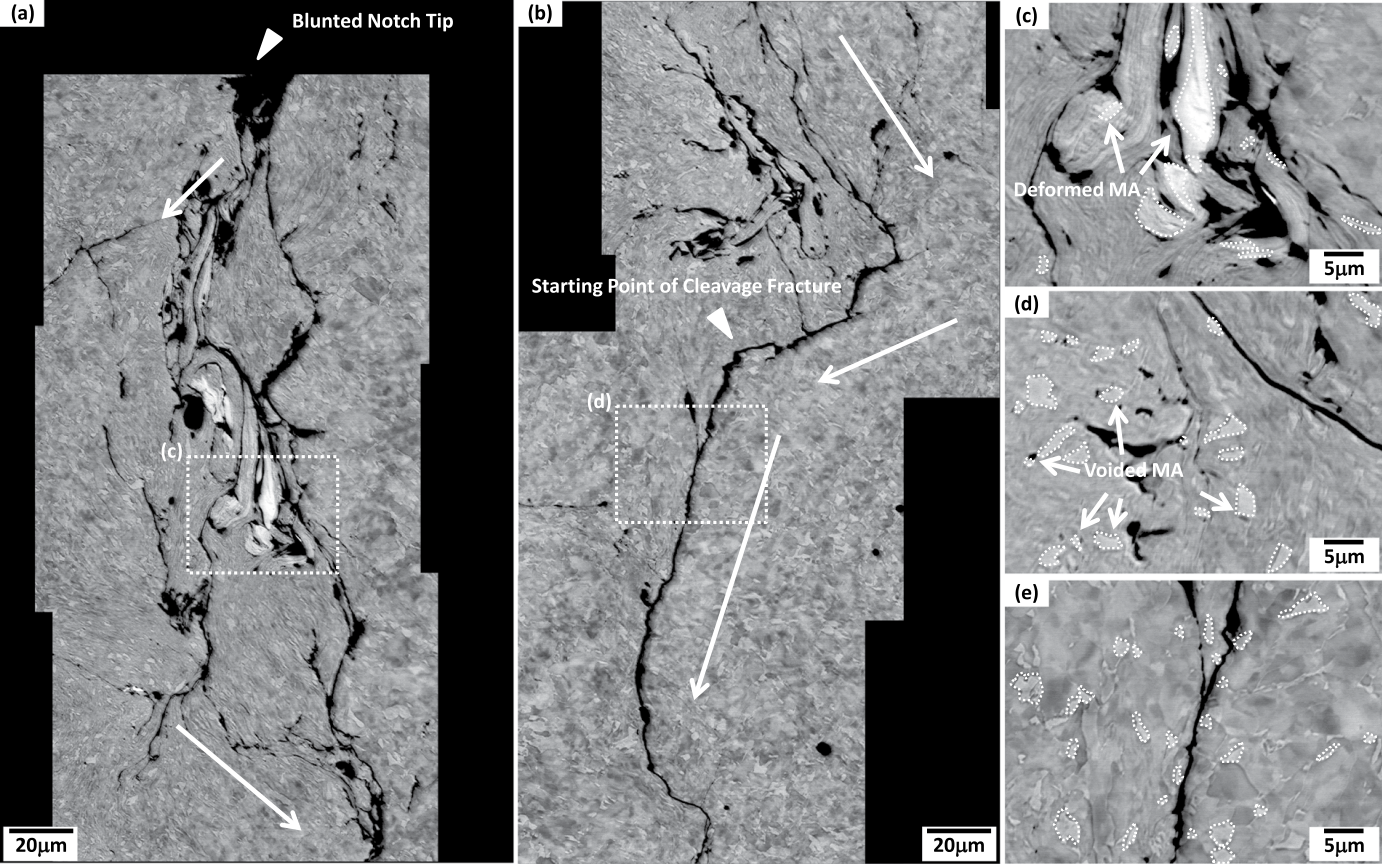
(**a**–**e**) SEM compo-images of the sharp-notch-tip area of the MM steel, together with blunted notch tip, starting point of cleavage fracture, and main crack propagation paths (white-arrow marks). In front of the blunted notch tip, there exists a largely deformed area as a number of cracks form, propagate, and meet together. MAs are deformed with the nearby matrix, and are interfacially debonded to form voids, which play a role in providing the crack propagation path, as shown in (**c**,**d**). After these cracks propagate along the loading direction to about 700 μm, a cleavage crack starts in a linear mode, as indicated by an arrow in (**b**).

Figure 5(a,b) shows SEM compo-images of the notch-tip area of the MM steel, together with blunted notch tip, starting point of cleavage fracture, and main crack propagation paths (white-arrow marks). A crack propagates in a shear direction (about 45 deg from the loading direction) from the notch tip, and then blunts at the arrow-marked point in Fig. 5(a). In front of the blunted notch tip, there exists a largely deformed area as a number of cracks form, propagate, and meet together (arrow marks). Figure 5(c) shows a compo-image of the dashed rectangular area (largely deformed area) of Fig. 5(a). Here in the compo-image, MAs are brightly shown, as indicated by white-dashed areas. These areas are largely plastically deformed, irrespective of kinds of microstructures. MAs are also deformed with the nearby matrix microstructures showing elongated shapes, although they are known to be hard and brittle^5–7,33^. Figure 5(d) shows a compo-image of another crack propagation area. Since MAs are known to promote the crack propagation by causing the stress concentration near their boundaries^2,34^, many MAs are interfacially debonded from the matrix to form voids, thereby providing the crack propagation path.

After these cracks propagate along the loading direction to about 700 μm, a cleavage crack starts in a linear mode (Fig. 5(b)). In the bending test specimen, the damaged layers are formed in a depth of 50 μm or less, while the cleavage crack starts to propagate at the position of about 700 μm distant from the notch tip. Before the cleavage crack propagation, the damaged layers show a ductile fracture behavior, like surrounding microstructures, which implies that they might not critically affect the cleavage crack propagation. According to the fractographic results^33^, ductile dimples are found in front of the notch tip, and changes to cleavage facets as the crack propagates further to the cleavage fracture starting point. Figure 5(e) shows a compo-image of a dashed rectangular area in Fig. 5(b). Since a cleavage crack passes mainly into the matrix by {100} cleavage planes, MAs might not play a role in cracking paths.

**Figure 6:**
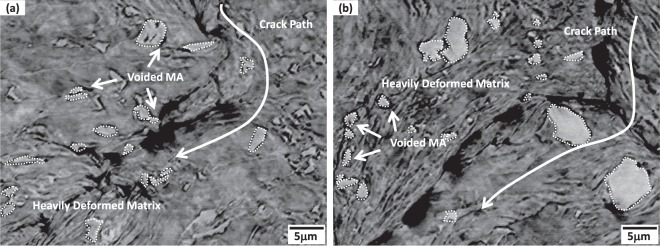
SEM micrographs of the sharp-notch-tip area of the (**a**) LM and (**b**) HM steels. As the crack propagation is blocked by the heavily deformed matrix regions, the crack path is frequently deviated, as indicated by curved white arrows.

Figure 6(a,b) shows the sharp-notch-tip area of the LM and HM steels. A crack initiates and propagates in front of the notch tip, while the matrix is largely deformed. As the crack propagation is blocked by the heavily deformed matrix regions, the crack path is frequently deviated, as indicated by curved white arrows. Inside the deformed matrix, very few cracks are found at MAs themselves, while MA/matrix interfaces are debonded to form voids, which indicates that the MA/matrix interfacial debonding is a main fracture mechanism in the ICHAZ.

### MA/matrix interfacial characteristics in ICHAZ

Average Vickers hardness data of QPF, GB, MA, and overall bulk are shown in Table S5. The Vickers hardness decreases in the order of MA, GB, and QPF. Since the difference in hardness between hard MA and softer QPF is larger than that between MA and GB, voided MAs might be formed more readily at the QPF area than at the GB areas, as shown in Figs 5(d) and 6(a,b). When MAs are located inside the QPF, they are more actively voided than those located inside the GB because of the stress concentration occurring during the deformation^33^. As the Mo content increases, the hardness values of QPF and GB, along with overall hardness values, increase, but this hardness trend breaks in the case of hardness of MA because the MA hardness of the MM steel is much lower than those of the other steels.

So as to find reasons why the debonding at MA/QPF interfaces prefers to the cracking of MAs, 3-dim. AP analyses were performed on MAs located at boundary and interior areas of QPF. Figure S2(a,b) shows SEM micrographs of an MA surrounded by nearby QPF grain boundaries or an MA located inside a QPF grain, respectively, in the MM steel. The MA located inside the QPF grain (interior MA) is generally smaller than that surrounded by QPF boundaries (boundary MA). A thick foil containing an MA was prepared by the ion-milling of outer areas.

**Figure 7:**
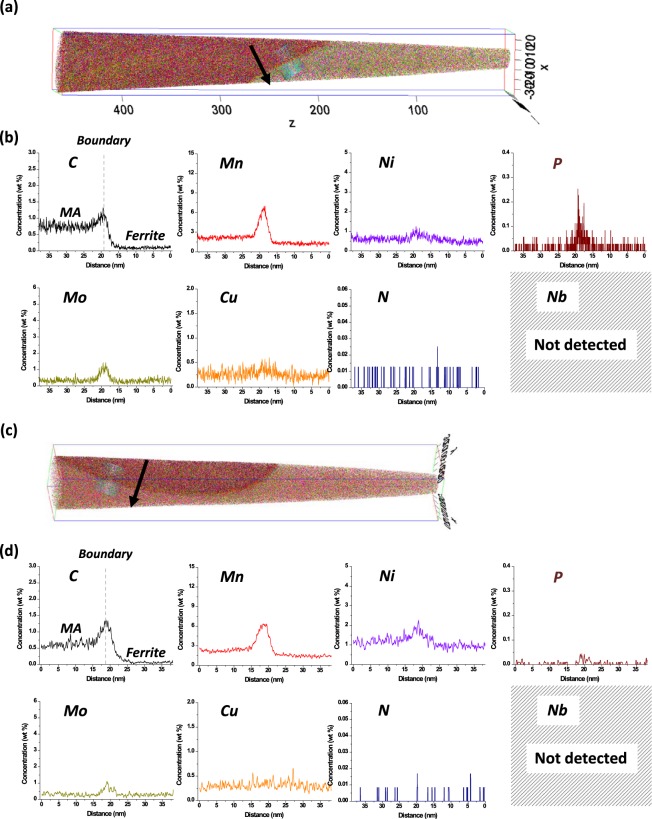
3-dim. atom probe (AP) image of a (**a**) boundary MA and (**c**) interior MA of the MM steel. The right side of the image is an MA, while the left side is a QPF, and these two microstructures are debonded by an interface. (**b**,**d**) shows line-profiles of various elements along a black arrow in (**a**,**c**). Major alloying elements such as C, Mn, Ni, P, and Mo are segregated at the MA/QPF interface.

A 3-dim. AP image of a boundary MA and line-profiles of alloying elements along a black arrow of the MM steel is shown in Fig. 7(a,b). According to the different C content in the AP image, the right and left sides of the interface are MA and QPF, respectively. The segregation of C is revealed from a peak at the MA/QPF interface. Peaks of Mn, Ni, P, and Mo are also shown, although the Ni peak is not high. Cu, N, and Nb seem to be hardly segregated.

Figure 7(c) shows an AP image of an interior MA and line-profiles of elements of the MM steel. Here again, a peak of C is shown at the interface (Fig. 7(d)), but the peak height seems to be lower than that of the boundary MA (Fig. 7(b)). The segregations of Mn, Ni, and Mo are also shown, like in the profiles of the boundary MA, whereas Cu and P are hardly segregated (Fig. 7(d)). When comparing these data with those of the boundary MA (Fig. 7(b)), the overall segregated amounts of major elements are lower in the interior MA.

Supplementary Fig. 3(a–f) shows line-profiles of alloying elements of boundary and interior MAs of the LM, MM, and HM steels. Peaks of C, Mn, and P are shown in the three steels, although those of Ni and Cu are not dominantly found. The peak of Mo of the HM steel is higher than that of the MM steel, whereas it is not found in the LM steel, which indicates that the Mo amount segregated at the MA/QPF interface increases with increasing Mo content of the steel nominal compositions (Supplementary Table 1). Here again, the overall segregation amounts in the interior MAs tend to be lower than those in the boundary MAs.

## Discussion

### Effects of microstructures on CTOD in CGHAZ

**Figure 8:**
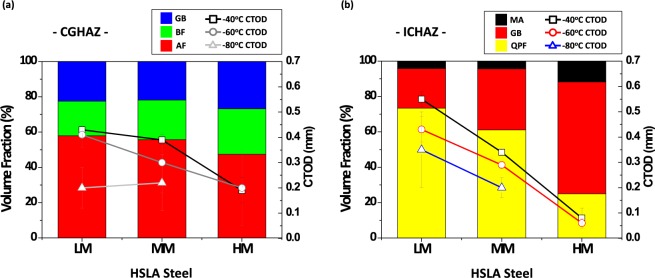
Volume fraction of microstructures (AF, GB, BF, QPF, and MA) and critical CTODs in the (**a**) CGHAZ and (**b**) ICHAZ of LM, MM, and HM steels. The CTOD increases with increasing AF fraction or with decreasing GB and BF fractions in the CGHAZ, while it increases with increasing OPF fraction or with decreasing GB and MA fractions in the ICHAZ.

The HAZ microstructure and resultant CTOD are influenced by the addition of Mo. Figure 8(a,b) shows volume fractions of microstructures in the CGHAZ and ICHAZ and CTODs of the LM, MM, and HM steels. In the CGHAZ, the CTOD increases with increasing AF fraction or with decreasing GB and BF fractions (Fig. 8(a)). The HM steel whose GB and BF fractions are highest shows the lowest CTOD. The prevention of GB and BF as well as the promotion of AF are desirable for enhancing the fracture toughness of the CGHAZ.

**Figure 9:**
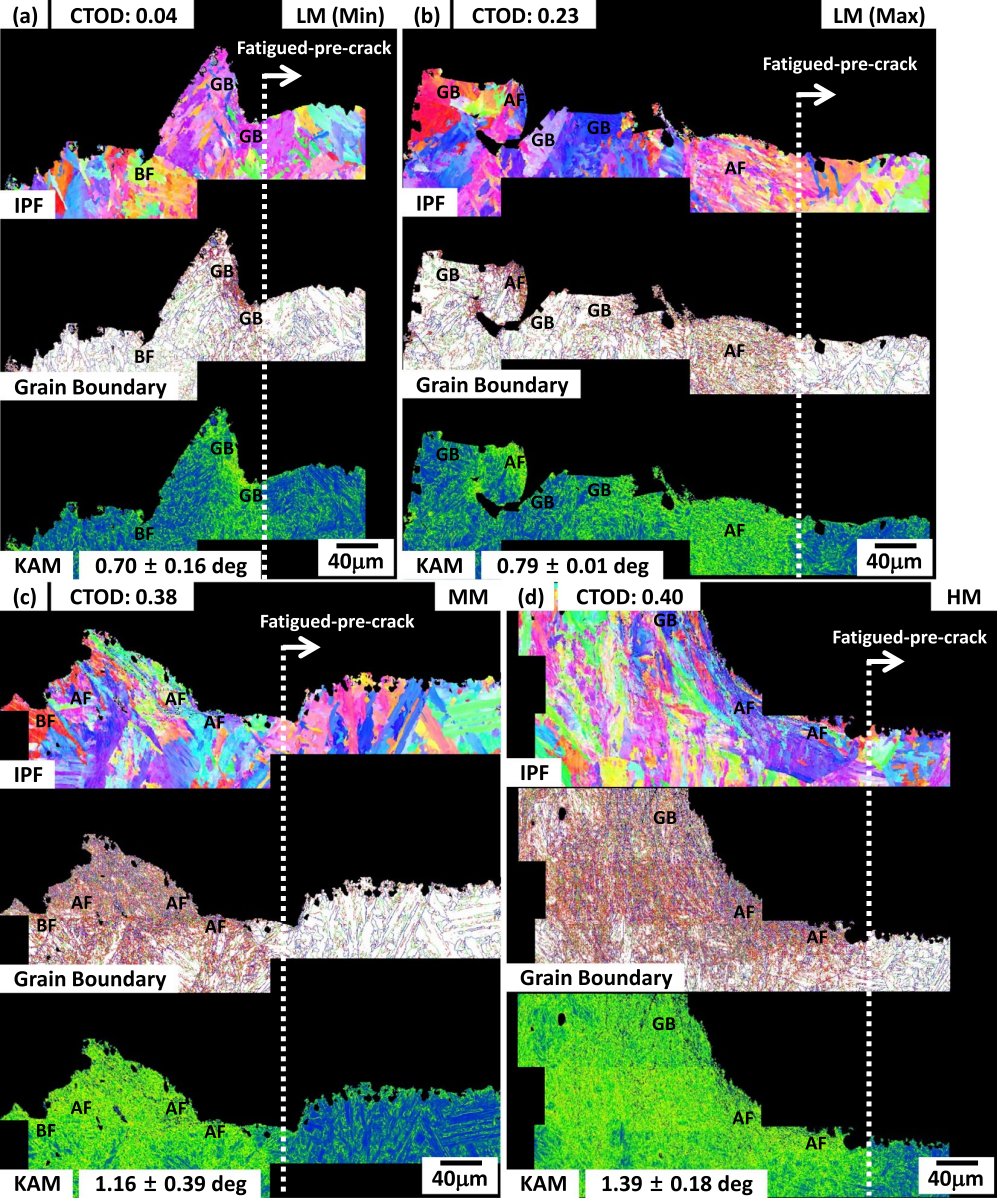
EBSD IPF, grain boundary, and kernel average misorientation (KAM) maps for two specimens having (**a**) minimum CTOD of LM steel and maximum CTODs of the (**b**) LM, (**c**) MM, and (**d**) HM steel. Fine AF grains and high KAM values are observed in front of the fatigued-pre-crack tip of both MM and HM steels as the crack-tip area is largely deformed.

As CTODs of the CGHAZ are largely varied even in the same steel and test temperature, fractured CTOD specimens of the LM steel were cross-sectioned, and the fatigued-pre-crack-tip areas were analyzed by the EBSD. Figure 9(a,b) shows IPF, grain boundary, and kernel average misorientation (KAM) maps for two specimens having minimum and maximum CTODs (0.04 mm *vs*. 0.23 mm at −60 °C). In the CTOD specimen having the minimum value (0.04 mm), the crack propagates across large packets of GBs located in front of the crack tip (Fig. 9(a)). KAM values are relatively low (average; 0.70 ± 0.16 deg) near the crack-tip area. In the specimen having the maximum CTOD, the crack propagates rather tortuously through highly deformable AF grains (Fig. 9(b)). Here, KAM values near the crack-tip area (average; 0.79 ± 0.01 deg) are higher than those of the minimum-CTOD specimen because of the active plastic deformation inside AF grains.

Figure 9(c,d) shows the EBSD results of the maximum-CTOD specimens of the MM and HM steels. In front of crack tip of both MM an HM steels, AF grains and high KAM values exist as the crack-tip area is largely deformed. The increased AF fraction results in the improvement of CTOD. These observations imply that the CTOD should be averaged after many specimens are tested because it is varied with crack-tip microstructures.

### Effects of MAs and their interfaces on CTOD in ICHAZ

In the ICHAZ, the CTOD decreases with increasing Mo addition, like in the CGHAZ (Fig. 8(b)). The HM steel shows the lowest CTOD because of the highest fractions of GB and MA (about 63% and 12%, respectively). The LM steel shows the highest CTOD because of the lowest fractions of GB and MA fractions. These CTOD results varied with the microstructural fractions are closely related to the Mo addition effect aforementioned in the section of ‘Microstructures of coarse-grained HAZ (CGHAZ)’. This is because the amount of carbon-rich austenite can increase by the Mo addition to cause the increase in MA fraction inside the ICHAZ^33,34^. The increase in QPF fraction favorably influences the CTOD because the fracture resistance of QPF is excellent^33^. Since hard MAs deteriorate the fracture resistance, they should be examined with the CTOD^5–7,33^. The HM steel whose MA fraction reaches 12% shows the lowest CTODs as the negative MA effect overrides the positive QPF effect.

**Figure 10:**
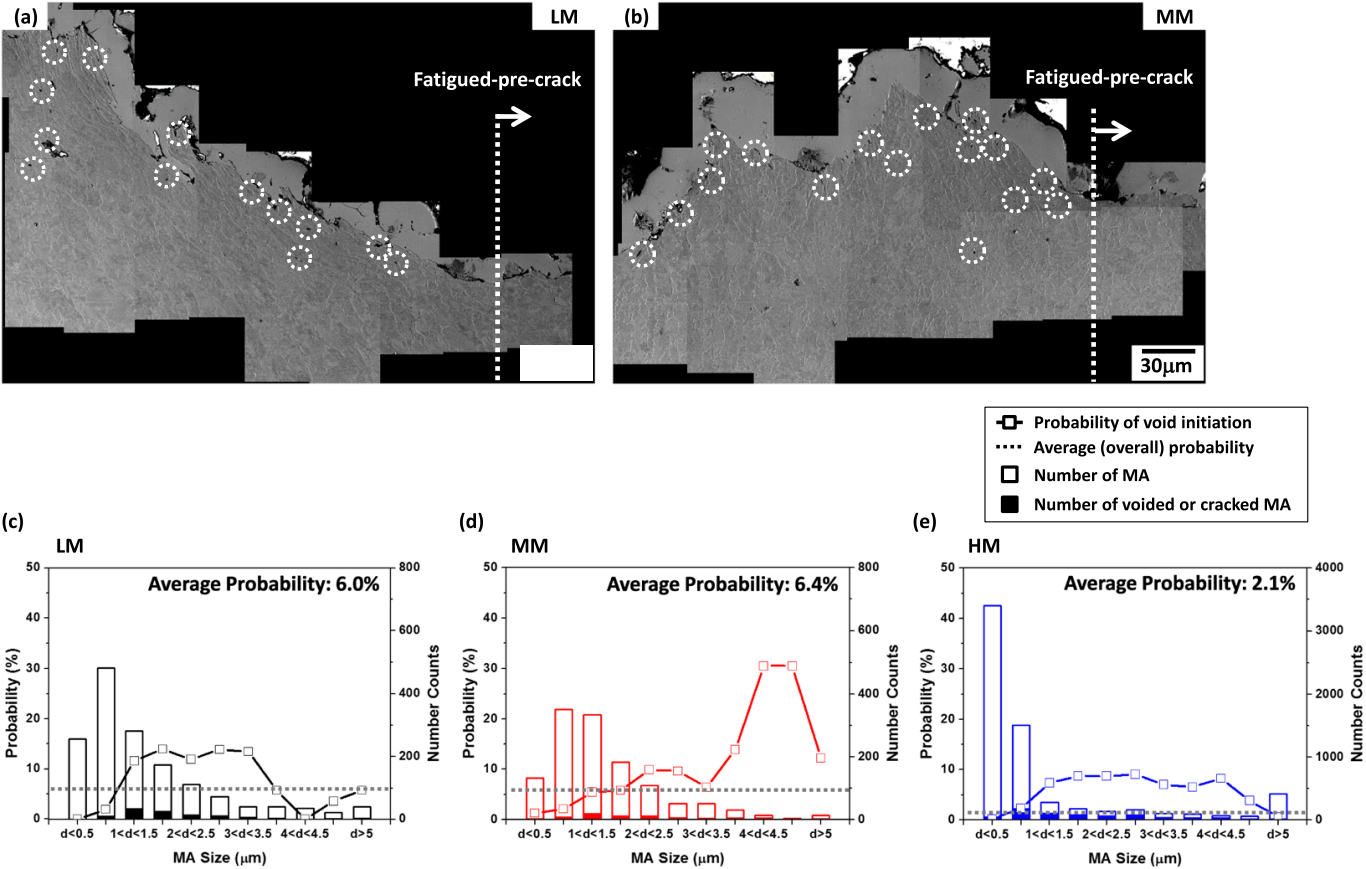
SEM micrographs of the cross-sectional area in front of the fatigued-pre-crack tip of the fractured CTOD specimens of the ICHAZ of the (**a**) LM and (**b**) MM steels tested at −60 °C. A considerable number of voids are found mainly at MA/matrix interfaces beneath the fracture surface, while some microcracks are formed by the cracking of MAs, as indicated by dotted circles. The probabilities of void initiation or cracking at MAs in the LM, MM, and HM steels are shown in (**c**–**e**). Here, the average (overall) probabilities are indicated by dotted lines.

When a number of MAs are distributed in the QPF or GB matrix, an effective size range of MA exists for the initiation of voids or microcracks^4,5^. SEM micrographs of cross-sectional crack-tip areas of the CTOD specimen of the LM and MM steels tested at −60 °C are shown in Fig. 10(a,b). Many voids are observed at MA/matrix interfaces as indicated by dotted circles, which implies that the interfacial void initiation is a major fracture mechanism, while a few microcracks formed by the MA cracking. The probabilities of void initiation or cracking at MAs were measured by counting voided or cracked MAs among total MAs, and the results as a function of size range of MA are shown in Fig. 10(c–e). In the LM steel, the probability is very low at the MA size of 0.5~1.0 μm, increases abruptly above 10% at the MA size of 1.0~1.5 μm, remains constantly to the MA size of 3.5 μm, and then decreases (Fig. 10(c)). Thus, MAs act as void initiation or cracking sites when their size range is 1.0~3.0 μm. The probability trends of the MM and HM steels are similar to those of the LM steel, although the probability in the MA size range of 4 μm or larger is high in the MM and HM steel (Fig. 10(d,e)). It is clearly visible that very small MAs can be hardly voided or cracked, whereas the probability of voided or cracked large MAs is varied with the steels.

In order to find reasons why the probability in the large MA size range of the MM steel is quite high, effects of MA/matrix interfacial debonding on crack initiation and propagation should be discussed. When MAs are populated, characteristics of MA/matrix interfaces might influence the CTOD more importantly than the MA fraction and size. This interfacial debonding works as a major mechanism, and is closely related with the segregation of alloying elements at MA/matrix interfaces. According to the AP line-profile data of boundary and interior MAs (Figs 7(b,d) and S3(a–f)), considerable amounts of C, Mn, Mo, and P are segregated at interfaces, while Ni and Cu are not seriously segregated. The segregated amounts of C, Mn, Mo, and P are much higher than the average amounts in nominal steel compositions (Table S1), which can weaken the interfacial bonding strength and result in the easy MA/matrix interfacial debonding. In particular, only a small amount of P is included in the steels (Table S1), but most of P might be segregated at the interface. Since the segregated Mo amount increases with increasing Mo content in the nominal steel composition (Fig. S3(a–f)), it is also expected that the interfacial debonding occurs readily in the high-Mo-containing HM steel, but the debonding trend is reversed. The average (overall) probability of interfacial void initiation of the HM steel is lower, particularly in the large size range of MA, than that of the MM steel (Fig. 10(d,e)). This implies that there is another factor affecting the void initiation.

The observation results of crack propagation of the notch-tip area of the MM steel (Fig. 5(a,c)) indicate that a number of MAs are well deformed with the nearby QPF matrix as the hardness of MA is much lower than that of the other steels (Table S5). The deformation of MAs is expected to be favorable for the improvement of CTOD. However, most of MA/matrix interfaces of the deformed MAs are debonded from the matrix to initiate voids, which provide easy crack propagation paths to deteriorate the CTOD, instead of the CTOD enhancement. This deformation-induced MA/matrix interfacial debonding is promoted when the hardness is low enough to be largely deformed with the matrix, while the segregation of elements (C, Mn, Mo, and P) helps to form interfacial voids (Fig. 7(a,b)). This result plausibly explains the high probability in the large MA size range (Fig. 10(d)). As a number of large MAs readily take part in initiating interfacial voids, the overall probability of interfacial void initiation of the MM steel is highest (Fig. 10(d)). Thus, the CTOD of the MM steel is much lower than that of the LM steel, although the volume fraction of MA is similar in the two steels.

The present study on fracture characteristics of complicated HAZ microstructures provides a good way for investigating correlations between Mo addition and CTOD. The measured CTOD data are plausibly explained by volume fractions of HAZ microstructures defined by microstructural classification methods. Particularly in the ICHAZ, the detailed microscopic analysis data including MA/matrix interfacial debonding due to segregation of alloying elements are outstanding ones for elucidating fracture mechanisms related with void initiation at MA/matrix interfaces. They also offer valuable ideas for HSLA steel designs and reliable evaluations of CTOD.

## Conclusions

Effects of Mo addition on microstructures and CTODs in the HAZs of the three HSLA steels were investigated in this study, and the correlation between them was explained by fracture mechanisms related with MA characteristics.

1. The CGHAZs of the three HSLA steels were composed of AF, GB, and BF, while the ICHAZs were composed QPF, GB, and MA. The volume fraction of AF in the CGHAZ decreased as the Mo content increased, while the GB and BF fractions showed an opposite trend of AF, which implied that the Mo addition prevented the formation of AF and promoted the formation of GB to decrease the CTOD. In the ICHAZ, the GB and MA fractions increased with the increased Mo content, whereas that of QPF decreased, and thus the CTOD decreased, like in the CGHAZ.
2. In the ICHAZ where many MAs were distributed in the QPF or GB matrix, an effective size range of MA existed for the initiation of voids or microcracks. Many voids were observed at MA/matrix interfaces, which indicated that the interfacial void initiation was a major fracture mechanism. According to the probability results of void initiation or cracking, MAs actively worked as void initiation or cracking sites when their size range was 1.0~3.0 μm.
3. The AP line-profile data of MAs indicated that considerable amounts of C, Mn, Mo, and P were segregated at MA/matrix interfaces, while Ni and Cu were not seriously segregated. The segregated amounts of C, Mn, Mo, and P are much higher than the average amounts in nominal steel compositions, which could weaken the interfacial bonding strength and result in the easy MA/matrix interfacial debonding. Thus, characteristics of MA/matrix interfaces might influence the CTOD more importantly than the MA fraction and size.
4. According to the observation results of crack propagation of the MM steel, many MAs were well deformed with the nearby QPF matrix as the hardness of MA was much lower than that of the other steels. The deformation of MAs was expected to be favorable for the CTOD, but most of MA/matrix interfaces of the deformed MAs were debonded to initiate voids, which provided easy crack propagation paths. Thus, the CTOD of the MM steel was lower than that of the LM steel, although the MA fraction was similar in the two steels.

## Method

### Fabrication of HSLS steels

The used steels were three HSLA steels, and their compositions are shown in Table S1. 100-mm-thick plates were produced by the commercial TMCP composed of reheating (at 1100~1200 °C), controlled-rolling (reduction ratio; 70~80%, finish rolling temperature; 760~800 °C above Ar_3_), accelerated-cooling to 300 °C (cooling rate; 4~10 °C/s), and air-cooling. They were tempered at 500~600 °C for 160 min to enhance the facture toughness.

### HAZ simulation and microstructural characterization

Rectangular bars (size; 10 × 10 × 60 mm, orientation; transverse) were heated to a peak temperatures (1350 °C or 720 °C to simulate welding heat cycles of the CGHAZ or ICHAZ, respectively), cooled to 500 °C (cooling rate; 15 °C/s), and further cooled to 200 °C (cooling rate; 7.5 °C/s) by a metal thermal-cycle simulator (MTCS, RnB, Korea). Microstructures of longitudinal-short-transverse (L-S) plane of weld-simulated specimens were examined by optical and scanning electron microscopes after the etching in a 1% nital solution. EBSD analysis was conducted by a field-emission SEM (Quanta 3D FEG, FEI Company, USA). Features of MA/matrix interfaces were analyzed by a 3-d AP (LEAP 3000X HR, Cameca) equipped with a focused ion beam (FIB, Quanta 3D, FEI, Netherlands).

### Hardness, tensile, and CTOD tests

Vickers hardness tests were performed to measure overall hardness values under a 500 g load. Vickers hardness values of microstructures such as QPF, GB, and MA in the ICHAZ were measured under a depth control by a nano-indenting machine (G200, Agilent, USA, tip; Berkovich tip, maximum depth; 1000 nm). After many nano-indentations were made, indentation points were confirmed by red dots in optical micrographs of Supplementary Fig. 4(a,b), and Vickers hardness values were averaged. Typical indented points of GB and MA are shown in Fig. S4(c,d). Dog-bone shaped plate-type tensile specimens whose gage length, width, and thickness were 25 mm, 6 mm, and 1 mm, respectively, were prepared along the longitudinal orientation, and were tested at room temperature by a universal testing machine (8801, Instron, USA, capacity; 100 kN, strain rate; 10^−3^ s^−1^). Pre-fatigue-cracked rectangular bars (10 × 10 × 60 mm) were prepared along the transverse-longitudinal (T-L) orientation for three-point-bend CTOD tests. They were tested at −40~−80 °C in accordance with the BS 7448 standard method^35^ by a testing machine (810, MTS, USA, capacity; 100 kN, cross-head speed; 0.02 mm/s).

## Electronic supplementary material

Supplementary Information

## Data Availability

The data that support the findings of this study are available from the corresponding author upon reasonable request.
