# Supplementary material for: Effects of Mo addition on crack tip opening displacement (CTOD) in heat affected zones (HAZs) of high-strength low-alloy (HSLA) steels
Source: Sci Rep. 2019 Jan 18;9:229. doi: 10.1038/s41598-018-36782-6 (PMC6338775; doi:10.1038/s41598-018-36782-6)
Supplement: Supplementary file 1 — Supplementary Information [file 41598_2018_36782_MOESM1_ESM.docx]

**Supplementary Information**

**Effect of Mo addition on crack tip opening displacement (CTOD) in**

**heat affected zones (HAZs) of high-strength low-alloy (HSLA) steels**

**Seok Gyu Lee ^a^, Bohee Kim ^a^, Woo Gyeom Kim ^b^, Kyung-Keun Um ^b^, Sunghak Lee ^a,*^**

***^a^ Center for Advanced Aerospace Materials***

***Pohang University of*** ***Science and Technology, Pohang 790-784, Republic of Korea***

***^b^ Steel Products Research Group 1***

***Technical Research Laboratories, POSCO, Pohang 790-785, Republic of Korea***

*Corresponding author: Sunghak Lee

[shlee@postech.ac.kr](mailto:shlee@postech.ac.kr)

Tel: +82-54-279-2140

Fax: +82-54-279-2399

**Supplementary Figures**


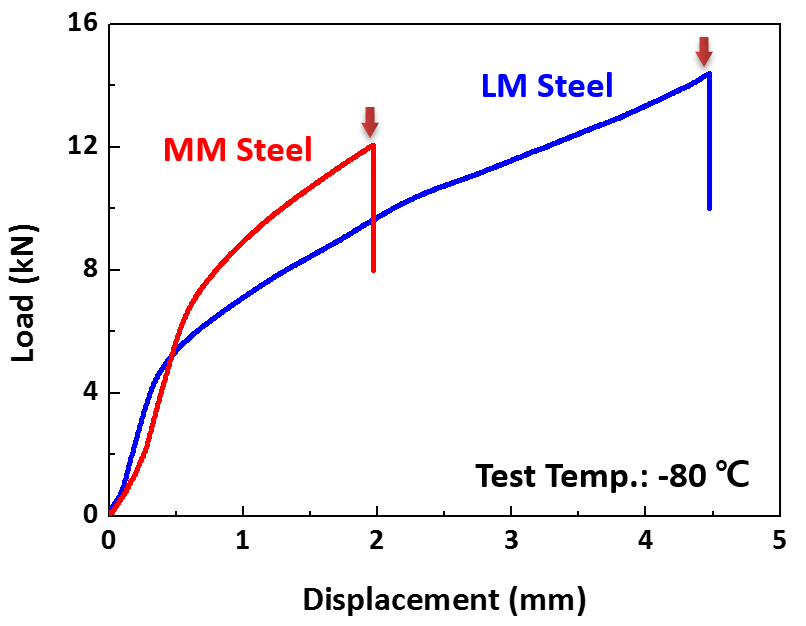


Supplementary Figure 1. Three-point bending load-displacement curve of the sharp-notch-introduced specimen (size; 10×10×60 mm, notch depth; 3 mm, T-L orientation, same to CTOD specimen dimensions) of the LM and MM steels tested at -80 °C. Based on these curves, the bending test was stopped before the specimen fracture, as marked by a red arrows.


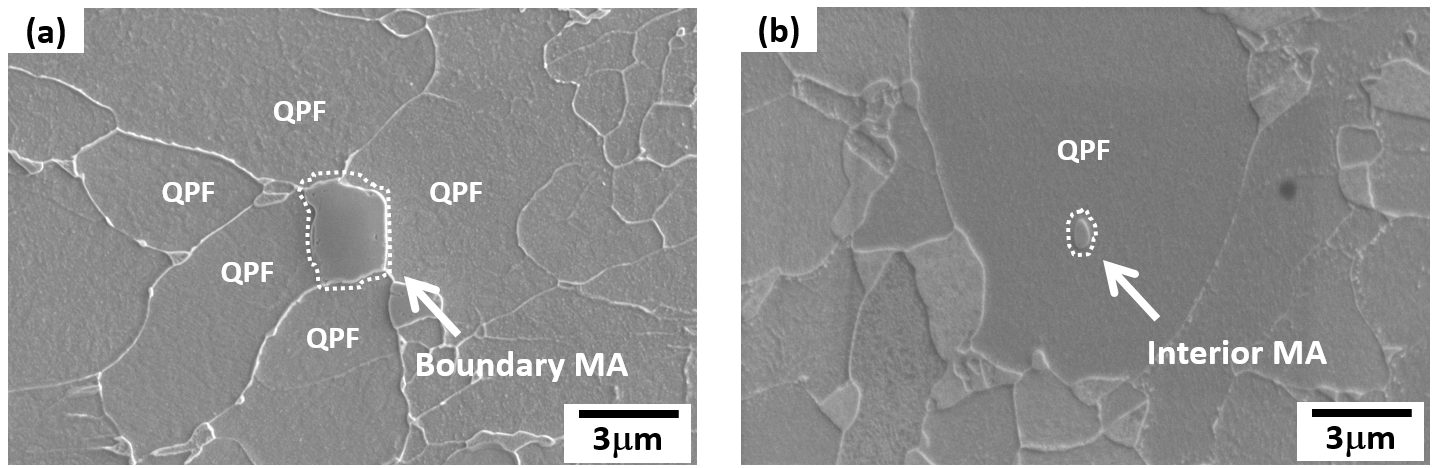


Supplementary Figure 2. SEM micrographs of an MA surrounded by nearby QPF grain boundaries or an MA located inside a QPF grain, respectively, in the MM steel. The MA located inside the QPF grain (interior MA) is generally smaller than that surrounded by QPF boundaries (boundary MA). A thick foil containing an MA was made by the Pt deposition and ion-milling of outer regions.


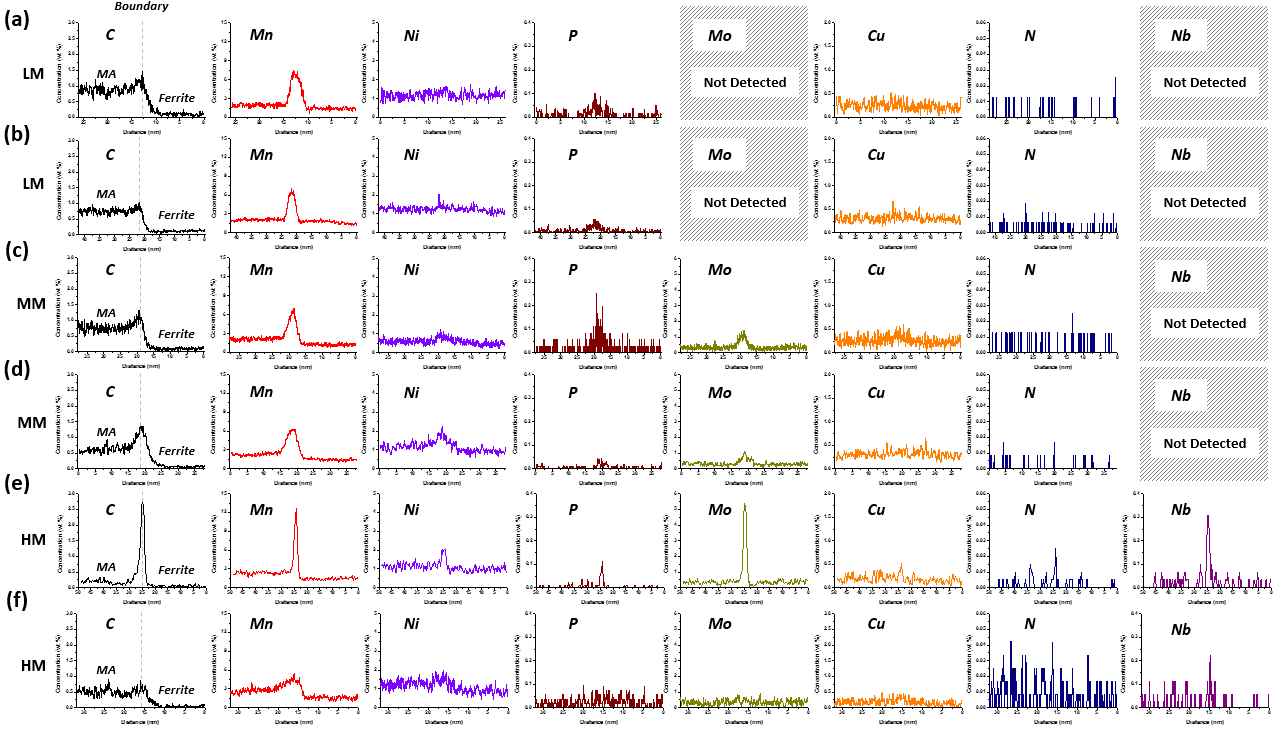


Supplementary Figure 3. 3-dim. AP line-profiles of alloying elements of boundary and interior MAs of (a,b) LM, (c,d) MM, and (e,f) HM steels. Peaks of C, Mn, and P are shown in the three steels, although those of Ni and Cu are not dominantly found. The peak of Mo is higher in the HM steel than that in the MM steel, whereas it is not found in the LM steel.


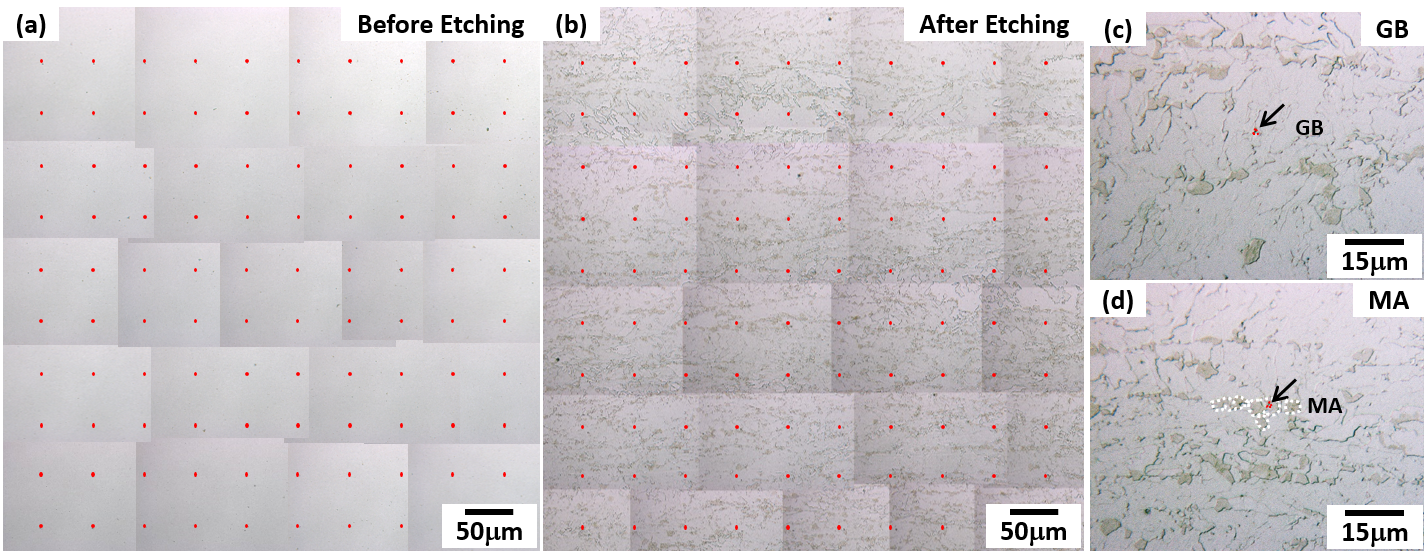


Supplementary Figure 4. Optical micrographs showing nano-indenting locations at QPF, GB, and MA (a) before and (b) after the nano-indentation. Red dots indicate nano-indentation points, from which average Vickers hardness values are obtained. Typical indented points of GB and MA are shown in (c,d).

**Supplementary Tables**

**Supplementary Table 1.** Chemical compositions of the three commercial S450~500-grade HSLA steels. (wt.%)

| Steel | C | Mn | Ni | Mo | Cr+Al+Ca | B+N | P+S | Si+Cu | Ti+Nb+V |
| --- | --- | --- | --- | --- | --- | --- | --- | --- | --- |
| LM | <0.07  <0.07  <0.07 | 1.2~2.0 | 0.8~1.8 | 0.002 | <0.04 | <0.004 | <0.008 | <0.7  <0.7  <0.7 | <0.03  <0.03  <0.03 |
| MM |  | 1.2~2.0 | 0.8~1.8 | 0.194 | <0.03 | <0.005 | <0.007 |  |  |
| HM |  | 1.2~2.0 | 0.8~2.2 | 0.350 | <0.04 | <0.004 | <0.007 |  |  |

**Supplementary Table 2.** Volume fractions of acicular ferrite (AF), granular bainite (GB), bainitic ferrite (BF), quasi polygonal ferrite (QPF), and martensite-austenite constituent (MA) in the simulated CGHAZ and ICHAZ of the three HSLA steels.

| Steel | HAZ | Volume Fraction (%) | | | | |
| --- | --- | --- | --- | --- | --- | --- |
|  |  | AF | GB | BF | QPF | MA |
| LM | CGHAZ | 57.9 ± 6.1 | 19.6 ± 5.6 | 22.5 ± 1.2 | - | - |
| MM |  | 55.7 ± 9.6 | 22.5 ± 1.7 | 21.9 ± 8.3 | - | - |
| HM |  | 47.4 ± 4.1 | 25.8 ± 2.4 | 26.8 ± 5.1 | - | - |
| LM | ICHAZ | - | 22.5 ± 3.7 | - | 73.3 ± 3.8 | 4.2 ± 1.1 |
| MM |  | - | 34.6 ± 1.1 | - | 61.0 ± 0.3 | 4.4 ± 0.5 |
| HM |  | - | 63.3 ± 2.5 | - | 25.0 ± 4.3 | 11.8 ± 1.9 |

**Supplementary Table 3.** Room-temperature tensile test results of the three HSLA steels.

| Steel | Yield Strength (MPa) | Tensile Strength (MPa) | Elongation (%) |
| --- | --- | --- | --- |
| LM | 447 ± 6.7 | 530 ± 7.2 | 34 ± 0.3 |
| MM | 485 ± 4.8 | 580 ± 0.2 | 29 ± 2.0 |
| HM | 559 ± 2.8 | 642 ± 3.2 | 27 ± 1.1 |

**Supplementary Table 4.** Critical CTODs measured at -40~-80 °C of the simulated CGHAZ and ICHAZ of the three HSLA steels. (unit: mm)

| Temperature | HAZ | LM Steel | MM Steel | HM Steel |
| --- | --- | --- | --- | --- |
| -40 °C | CGHAZ | 0.43 ± 0.03 | 0.39 ± 0.02 | 0.19 ± 0.05 |
| -60 °C |  | 0.41 ± 0.05 | 0.30 ± 0.12 | 0.20 ± 0.15 |
| -80 °C |  | 0.20 ± 0.08 | 0.22 ± 0.11 | - |
| -40 °C | ICHAZ | 0.55 ± 0.03 | 0.34 ± 0.01 | 0.08 ± 0.04 |
| -60 °C |  | 0.43 ± 0.05 | 0.29 ± 0.01 | 0.06 ± 0.02 |
| -80 °C |  | 0.35 ± 0.15 | 0.20 ± 0.04 | - |

**Supplementary Table 5.** Vickers hardness values of quasi-polygonal ferrite (QPF), granular bainite (GB), and martensite-austenite constituent (MA) and overall bulk in the simulated ICHAZ of the three HSLA steels. (unit: VHN)

| Steel | QPF | GB | MA | Overall Bulk |
| --- | --- | --- | --- | --- |
| LM | 176 ± 12.9 | 207 ± 7.9 | 277 ± 50.2 | 174 ± 0.7 |
| MM | 179 ± 14.3 | 215 ± 12.8 | 231 ± 48.3 | 181 ± 2.7 |
| HM | 198 ± 4.0 | 229 ± 21.5 | 327 ± 45.4 | 210 ± 3.4 |
